# Psychosomatic response to acute emotional stress in healthy students

**DOI:** 10.3389/fphys.2022.960118

**Published:** 2023-01-09

**Authors:** Esther García Pagès, Adriana Arza, Jesús Lazaro, Carlos Puig, Thais Castro, Manuel Ottaviano, Maria Teresa Arredondo, Maria Luisa Bernal, Raúl López-Antón, Concepción De La Cámara, Eduardo Gil, Pablo Laguna, Raquel Bailón, Jordi Aguiló, Jorge Mario Garzón-Rey

**Affiliations:** ^1^ Universitat Autònoma de Barcelona, UAB, Barcelona, Spain; ^2^ École Polytechnique Fédérale de Lausanne, EPFL, Lausanne, Switzerland; ^3^ Universidad de Zaragoza, UZ, Zaragoza, Spain; ^4^ Centro de Investigación Biomédica en Red de Bioingeniería, Biomateriales y Nanomedicina, CIBER-BBN, Madrid, Spain; ^5^ Life Supporting Technologies, Universidad Politécnica de Madrid, UPM, Madrid, Spain; ^6^ Omya Clariana S.L.U., OMYA, Barcelona, Spain

**Keywords:** stress, trier social stress test, cortisol, heart rate variability, multimodal biomarkers, pulse arrival time (PAT)

## Abstract

The multidimensionality of the stress response has shown the complexity of this phenomenon and therefore the impossibility of finding a unique biomarker among the physiological variables related to stress. An experimental study was designed and performed to guarantee the correct synchronous and concurrent measure of psychometric tests, biochemical variables and physiological features related to acute emotional stress. The population studied corresponds to a group of 120 university students between 20 and 30 years of age, with healthy habits and without a diagnosis of chronic or psychiatric illnesses. Following the protocol of the experimental pilot, each participant reached a relaxing state and a stress state in two sessions of measurement for equivalent periods. Both states are correctly achieved evidenced by the psychometric test results and the biochemical variables. A Stress Reference Scale is proposed based on these two sets of variables. Then, aiming for a non-invasive and continuous approach, the Acute Stress Model correlated to the previous scale is also proposed, supported only by physiological signals. Preliminary results support the feasibility of measuring/quantifying the stress level. Although the results are limited to the population and stimulus type, the procedure and methodological analysis used for the assessment of acute stress in young people can be extrapolated to other populations and types of stress.

## 1 Introduction

The definition of stress remains ambiguous despite the efforts made in its study. In fact, post-traumatic stress disorder (PTSD) is the only one described in the medical diagnostic manuals: DSM-V (American Psychiatric Association, 2013) and CIE-10 (Brämer, 1988). Beyond PTSD, the most cited definition of stress was provided by H. Selye within the framework of what he called the “General Adaptation Syndrome”. Selye defines stress as “The non-specific response of the body to the request for change” or as “a complex pattern of reactions that usually has psychological, cognitive and behavioural components” (Selye, 1950).

Stress was declared a global epidemic by the World Health Organization in the 21st century (Bakker and Holenderski, 2012). In addition, the prevalence of stress has been linked to negative effects on both psychic and physical health (Cohen et al., 1997). However, the level and/or duration required for its transformation into a pathogenic agent is unknown. Chronic stress arises when the stress response is prolonged and a rearrangement of the homeostatic equilibrium may take place. These new equilibrium conditions can affect the subject’s wellbeing and even negatively affect their health. Again, depending on the stressor, the time and the subject himself, the equilibrium may never be reached again and the subject’s health may be severely affected. In this case, we talk about post-traumatic stress disorder. On the opposite side, acute stress arises when the stress response remains for minutes or a few hours affecting the homeostatic equilibrium.

Among the strategies reported in the literature for stress measurement, three different approaches can be highlighted (Hellhammer et al., 2010): the assessment of the stress stimulus, the measurement of psychological perception of the stress stimulus and the measurement of the physiological changes under a stress stimulus.

The first strategy is based on the information gathering of events that occur in an individual’s lifetime, assessing the effect they produce and understanding that the accumulation of stressful events generates a higher affectation level. This strategy assumes that any event similarly affects all individuals. The Holmes and Rahe Stress Scale (Holmes and Rahe, 1967) is a well-known instrument based on this strategy, where a list of possible events is associated with a score.

The second approach focuses on the perception that an observer has of a subject exposed to a stimulus. This approach usually uses questionnaires. Clearly, this method is subjective and comparing subjects from different observers is difficult. Another alternative within this strategy is the self-perception of the subject, on behalf of the own psychosocial environment. For example, the Derogatis Stress profile (Derogatis, 1987) has 77 questions rating the stress level based on personality traits and the emotional response.

The third approach is based on the measurement of the physiological response of the subject to a stress stimulus, namely caused by the autonomic nervous system (ANS) and hypothalamic-pituitary-adrenal axis (HPA) activation. This response can be measured using anatomical, endocrine, haematological, electrical, thermal, immune and genetic markers. Although the physiological approach seems to be the most objective, it can be invasive, not very specific and may leave the subjective perception of the stress aside.

In any case, in order to characterise the stress response, a stress stimulus is needed. It could be based on physical elements, social interaction, emotional reactions or cognitive tasks. For example, sinking the hand or foot in cold or hot water are types of stimuli known as the Cold Pressor Test and the Warm Water Test. Similarly, Project ES3 (Aguilo et al., 2015) has studied the response to stress caused by high ambient temperatures with high humidity simulated in a climatic chamber (Garzon-Rey et al., 2016). Exposure to videos of corneal transplant surgeries has made it possible to characterise cortisol and alpha-amylase as stress biomarkers (Takai et al., 2004).

The Trier Social Stress Test (TSST) is a widely accepted protocol for robustly inducing moderate acute stress in human populations using different stimuli. TSST has made it possible to study the relationship between psychological and physiological responses in recent decades (Kudielka et al., 2007; Sequeira et al., 2021). Perhaps its usefulness lies in the simulation of a combination of a memory task, a social interaction event followed by an arithmetic task.

An increase in salivary, plasma and serum cortisol levels has been reported on TSST application, as well as in the androgen precursor dehydroepiandrosterone (DHEA) and its metabolite dehydroepiandrosterone-sulphate (DHEA-S). Higher levels of adrenaline (Gold et al., 2004) and salivary α-amylase (Het et al., 2009) have also been reported following the TSST. Furthermore, the immune system is sensitive to stress and has been linked to the HPA axis activation. Pro-inflammatory cytokines are able to activate the HPA axis and alter glucocorticoid secretion, as well as glucocorticoids can affect cytokine production. Pro-inflammatory cytokine interleukin-6 (IL-6) levels can increase by 50% in response to TSST and remain elevated 20 min after stress exposure. Elevated levels of IL-6 following the TSST are associated with a higher heart rate during the arithmetic task and a lower cortisol response (Allen et al., 2014).

Within the chain of secretions caused by the activation of the HPA axis are arginine vasopressin (AVP) and prolactin. AVP has a small molecular size and a short average lifetime that makes it difficult to measure. However, the concentration of Copeptin derived from the same AVP precursor has been proposed as a more stable surrogate, although its physiological interpretation is unknown (Urwyler et al., 2015). On the other hand, it is well established that prolactin secretion and plasma glucose levels are affected by stress. An elevation of their levels in response to different types of psychological stimuli in humans has been described and the intensity of the response depends on the intensity of the stressful stimulus (Armario et al., 1996).

Additionally, for a non-invasive measure of the ANS response to stress, Heart Rate Variability (HRV) derived from the electrocardiogram (ECG) signal is commonly used (Task Force, 1996). The psychophysiological factors that influence the stress level can be reflected in the heart rate (HR) and its variations, HRV (HR variability). These are both mediated by the ANS in its sympathetic and parasympathetic branches, which is reflected in the low-frequency (LF) and high-frequency (HF) components, depending on whether one or the other branch was predominant, respectively. A meta-analysis shows that there is a reduction in the HRV, an increase in the LF band parallel to a reduction in the HF band, and/or an increase in the LF/HF quotient, thereby reflecting an increase in sympathetic stimulation and parasympathetic inhibition during a stressful event (Kim et al., 2018).

Photoplethysmography (PPG) is an uncomplicated and inexpensive optical measurement alternatively used for heart rate monitoring purposes among others, more and more present in wearables nowadays. This signal can be analysed in order to extract similar information to the HRV, that is, the Pulse Rate Variability (PRV). Moreover, the PPG signal together with the ECG signal provides useful information about the time lapse between the two, the Pulse Arrival Time (PAT), which is inversely proportional to the pulse wave velocity, which is in turn associated with arterial stiffness and cardiovascular output. Therefore, a decrease in PAT is related to an increase in blood vessel resistance and cardiovascular output, as well as being inversely related to blood pressure (Wong et al., 2011).

There are also reports of an increase in the respiratory rate, RR, and its variability, RRV (Vlemincx et al., 2011; Milagro et al., 2017), and even an alteration affecting the synchronisation between them (Lackner et al., 2011) as a response to the action of a stressor. Increased electrodermal activity (EDA) has also been observed during TSST (Jezova et al., 2004). Finally, in other studies a relationship has been established between stress and blood pressure (BP), the electrical characteristics of the skin, and skin temperature (T) (De Santos-Sierra et al., 2011; Vinkers et al., 2013; Allen et al., 2014; Haufe et al., 2014).

To sum up, the stress response is mainly generated through the hypothalamic centres and the hypophysis in order to simultaneously activate the ANS and the HPA axis, although with different timings, being the former faster than the latter. Therefore, the main goal of this study is to establish a methodology to facilitate the objective, non-invasive and continuous measurement of stress through the study of changes induced by these two pathways. To that end, two steps were needed which constitute the two specific objectives of the present paper.

The first objective is to generate a Stress Reference Scale (SRS) as a linear combination of psychometric tests and biochemical variables allowing to better differentiate between stressed and relax states. However, since the SRS temporal resolution is limited and it is invasive, our second objective is to establish a methodology that would allow the objective, non-invasive and continuous measurement of stress, generating a model from physiological features alone that could lead to a more practical and extensive application.

For this purpose, the present paper reports an experimental study performed on healthy university students, designed to guarantee a synchronous and concurrent measure of psychometric tests, biochemical variables and electrophysiological features related to acute stress using a modification of the TSST to induce levels of stress based on different stimuli.

## 2 Methodology

### 2.1 Participants

Students from the University of Zaragoza, the Technical University of Madrid and the Autonomous University of Barcelona, Spain were recruited consecutively using advertisements on websites. Through the web portal of the project, a participation survey was carried out among interested students. In the survey, the objective of the project and the conditions of participation were explained in a general way, as well as the inclusion and exclusion criteria.

The conditions for inclusion in the study were to be a healthy student aged between 19 and 30 years old and a BMI lower than 30, while any participant with psychopathology, regular consumption of psychotropic substances, or when the baseline stress level was greater than 70% on the visual analogue scale, was excluded. Moreover, they could not consume tobacco, alcohol or stimulants of the Nervous System (caffeine, theine, taurine, etc) for at least 8 h prior to the study.

Those interested who meet the inclusion criteria were selected, up to a maximum of 60 participants per site and they were contacted to indicate the date and place of the first session. All participants signed an informed consent form approved by the ethics committee of each institution.

### 2.2 Experimental protocol

The proposed experimental design is an observational and transversal study, in which each individual is compared with himself or herself in a relaxed condition and in an acute stress condition in two measurement sessions. Each session took place on different days one or 2 weeks from each other, at the same time of day normally 2 h after waking up. Both sessions started with a relaxation period for 10 min with the help of an autogenous relaxing audio to prevent the arriving state from interfering with the study, named baseline relax (BR) and baseline stress (BS).

At the end of each session, a nurse took a blood sample. In addition, two samples of saliva were extracted per session at the end and at the beginning, which for the stress stage, it was 25 min before the end of the session, which corresponds to the end of the baseline stage.

#### 2.2.1 Relax session

During the first session, the relevance of the study and the protocol were explained to the participant and the informed consent was signed. Then, sensors were placed and clinical and socio-demographic data were documented. Afterwards, the subject was left alone in the room, with dimmed lights, in a comfortable position and with an audio for autogenous relaxation for 25 min following Schultz’s method (Stetter et al., 2002). After the BR stage, the first saliva sample was taken. The next 25 min were designated the Relax (R) stage, and, after that, saliva and blood samples were collected and the psychometric tests administered.

#### 2.2.2 Stress session

During this second session, after the 10 min BS stage, the first saliva sample was collected and the stress protocol began, which included three short stories, a memory test, a stress anticipation period, the video presentation and an arithmetic task.

The memory test was carried out with an interviewer and the subject alone in a room and consists of two stages. Within the first stage, referred to as Story Telling (ST), the subject was asked to listen carefully to three stories, which he/she will have to reproduce with the greatest number of details during the second stage, the Memory Test (MT), with a 30 s time limit for each story. The MT stage was video recorded.

Under the pretext that the video will be evaluated by experts to assess memory performance, the subject remains alone in the room without knowing how long he/she will be there. This stage aims to increase the state of anxiety before the next stage, and it was labelled as Stress Anticipation (SA).

After 10 min, the interviewer and an audience of at least three people entered the room to make the public presentation of the video segments (VD). The recorded videos of the subject were interspersed with videos from the control segments, which are equivalent video segments where a dummy participant responds to the same stories correctly. This unknown subject was matched in age and gender for each participant.

Finally, during the arithmetic task (AT) the subject was asked to count down from 1022 to 0 in steps of 13. If he/she made an error, he/she had to start over from the beginning (i.e., 1022). The subject had 5 min to complete the task, even though the subject was not expected to finish the task. After that, the second saliva sample and blood draw took place along with the psychometric tests.

**FIGURE 1:**
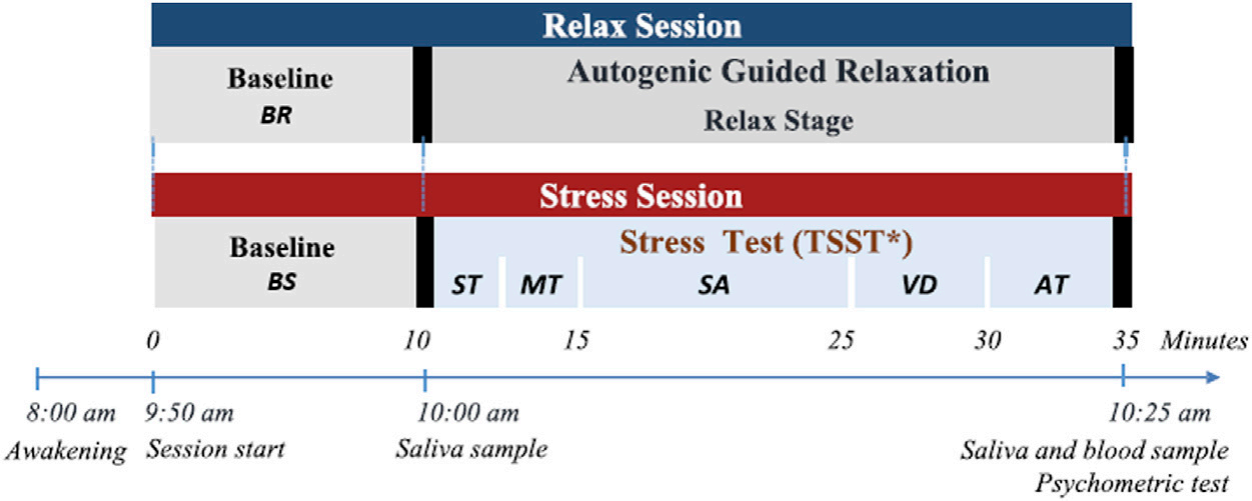
Experimental design sessions. BR: Baseline relax. BS: Baseline stress. ST: Story Telling. MT: Memory test. SA: Stress anticipation. VD: Public video display. AT: Arithmetic Task.

Figure 1 summarises the process followed during the two sessions.

### 2.3 Outcome measures

#### 2.3.1 Psychometric tests

The psychometric tests that were self-administered are the Perceived Stress Scale, the Visual Analog Scale, and the State-Trait Anxiety Inventory. All tests are validated, supported, well documented by the medical world and have a contrasted and accepted Spanish language version. The Symptomatic Stress Scale, designed at the Clinical Hospital of Zaragoza in the Project ES3 framework, was also administered.

The two sessions took place on different days to prevent the learning effect or memorisation of the questionnaires, which could invalidate the psychometric test results.

The State-Trait Anxiety Inventory (STAI) was built by C. Spielberger, R.L. Gorsuch, and R.E. Lushene, and consists of 40 questions related to the subject’s introspection about his feelings of discomfort, worry, tension and stress (Takai et al., 2004). The scale aims to measure two components: a trait component (STAIt, Trait Anxiety Inventory) that would quantify the relatively stable individual differences in responding to situations perceived as threatening or the tendency to perceive situations as more threatening. The second component refers to the state (STAIs, State Anxiety Inventory) in a transitional period characterised by a feeling of tension, apprehension and increased activity of the ANS (Spielberger, 2010). Each of the components is measured through a subset of 20 questions out of 40 on the scale. The STAIt and STAIs have total ranges from 0 to 60, with a higher score corresponding to higher detected anxiety. The Cronbach’s alpha of the scale, both in its trait and state component, is greater than 0.9 (Guillen-Riquelme & Buela-Casal, 2011).

The Visual Analog Scale (VAS) is a simple method to detect changes in mood and subjective perceptions (Monk, 1989). It consists of a single question: In general, where would you say your stress level is at this moment? Being 0 = no stress at all and 100 = absolutely stressed. The use of the VAS has shown high internal consistency in the measurement of transient and subjective psychological states (Cronbach’s alpha range: 0.84–0.96) (Abend et al., 2014).

The Perceived Stress Scale (PSS) was proposed in the late 20th century by Sheldon Cohen and his colleagues with a set of 14 items that were later reduced to 10. This scale assesses the extent to which life situations are assessed as stressful based on feelings and thoughts over the past month. Intrinsically, it considers the influence of how well the subject can handle a stressful situation given his or her coping resources, which ultimately offers a characterization of a person’s trait (Cohen et al., 1983). A higher score corresponds to a higher level of perceived stress. The short European Spanish version of 10 items demonstrated adequate reliability with Cronbach’s alpha 0.82 (Remor, 2006).

The Symptomatic Stress Scale (SSS) is a psychometric test designed on behalf of the ES3 Project (Aguilo et al., 2015) by the psychiatry research group of the Hospital Clínico de Zaragoza. It tries to evaluate the effect of the stressful stimulus on the subject from the somatic and psycho-cognitive symptoms that the subject perceives of himself (Table A1). The scale is of the Likert type and consists of 20 questions.

#### 2.3.2 Biochemical variables

The biochemical variables considered in the present study are the concentrations of hormones, enzymes or molecules, analysed in blood or saliva samples that offer information about the hypothalamic-pituitary-adrenal axis (HPA) and the autonomic nervous system (ANS).

The biochemical variables measured in saliva were cortisol and α-amylase, whereas the biochemical variables measured in blood were prolactin, copeptin, and glucose.

It was proposed to hold the morning sessions because of the circadian rhythm of cortisol and α-amylase, specifically the sessions were scheduled in the morning (09:45–10:45 h), 2 h after waking up. Moreover, it helps to have control of the foods consumed by the subjects and in the awakening, two factors that could invalidate the biochemical variables. All subjects were asked to avoid coffee and tea before the session and refrain from exercising or drinking alcohol in the last 24 h.

The saliva collection was performed in Salivette tubes following the manufacturer’s recommendations (Sarstedt AG & Co., Nümbrecht, Germany). Chewing the cotton swab provided with the tube and avoiding swallowing saliva for 1 min to obtain an adequate amount of sample.

Subsequently, the sample was preserved on ice until it was centrifuged in the laboratory (15 min at 1315 g, 4°C) to separate the saliva from the cotton swab. Then it was aliquoted in duplicate in Eppendorf tubes with the subject identifying data, type of session and date. The tubes were kept frozen at −20°C until processing.

The concentration of cortisol and α-amylase in saliva were quantified through commercial kits. A commercial immunoassay technique (Salimetrics, State College, PA, United States) was used for cortisol and a kinetic enzyme assay was used for α-amylase (Salimetrics, State College, PA, United States), capable of measuring the activity of the enzyme in international units/ml of saliva (U/ml).

The extraction of blood was carried out in two tubes: the first with EDTA anticoagulant and the second with gel separator. Both were preserved on ice until centrifuged in the laboratory at 3000 rpm for 10 min. The plasma and serum were then aliquoted separately in tubes with the identifying data of the subject, type of session and date. The tubes were kept frozen at −20 °C until processing at the Biomedical Diagnostic Center of the Hospital Clínico de Barcelona by molecular absorption and immunoassay spectrometry techniques to determine the concentration of prolactin, copeptin and glucose.

#### 2.3.3 Physiological signals

Physiological signals such as electrocardiogram (ECG), photopletismography (PPG), electrodermal activity (EDA), respiration (Resp) and skin temperature (T) change with stress. Many physiological features extracted from them are reported to vary with stress in the literature, but all of them are non-specific. We simultaneously recorded all these signals and processed them separately.

The final set of signals selected due to their relation to the stress response are the following: ECG of three orthogonal leads in the chest (1000 Hz), PPG in the middle finger of the non-dominant hand and the temple (250 Hz), EDA between the second phalanx of the ring finger and the index finger of the non-dominant hand (250 Hz), ST in the little finger and non-dominant cheek (sampling frequency 250 Hz) and the RR with a chest band (250 Hz). All the signals were recorded using the Medicom 83 system, ABP-10 module (Medicom MTD Ltd, Russia).

For the signal processing and feature extraction in 1-min windows, the MATLAB software was used. The beat detection in the ECG signal was performed using the wavelet transform-based algorithm reported in (Martinez, et al., 2004). After beat detection, the existence of ectopic or non-normal beats was verified using the algorithm reported in (Mateo et al., 2003). The algorithm looks for sudden changes in the time between beats and proposer an interpolated beat where non-normal beats are detected.

The conditioning of the photoplethysmography signal was carried out with a low-pass FIR filter with cut-off frequency 35 Hz and order 50 and a high-pass FIR filter with cut-off frequency of 0.3 Hz and order 5000. In order to detect artifacts in the signal, the average power was estimated from the filtered signal in windows of 1 s displaced every 0.5 s. The power was calculated as the variance of the signal every 0.5 s in the previous second and it was linearly interpolated over the time vector of the filtered signal. An artifact was defined as a signal point whose associated average power was greater than the mean plus three standard deviations of the average power associated with the signal of the immediately previous minute. Given that the PPG signal records present abrupt changes in amplitude of long duration or that they can be considered sections of low signal quality, the detection of this type of artifacts was carried out based on Hjorth parameters in windows of 2 seconds displaced every half-second using the algorithm described in (Gil et al., 2009). Finally, the detection of beats or pulses in the signal without artifacts was performed using the strategy reported in (Lázaro et al., 2014). The pulse detector algorithm consists of two phases: a linear filtering transformation, and an adaptive thresholding operation. Finally, the generation of pulse rate signal for the PRV analysis, the fiducial point of each pulse in PPG was defined as the one in which the amplitude has reached the 50% of its maximum. Once the pulses were detected, the existence of ectopic or non-normal beats was also verified.

Subsequently, the pulse arrival time was estimated as the time between the beats detected on the ECG and the pulse detected by the PPG on the finger.

The skin conductance signal was visually inspected to delete motion artefacts and linearly interpolated. The 1-min windows with interpolated segments larger than 25% were discarded and the signal was then resampled at 4 Hz. For the EDA processing and decomposition, we applied the convex optimization model (cvxEDA). In parallel, the signal was filtered with a band pass FIR filter with order 500 and a cut-off frequency of 0.01 Hz and 0.9 Hz for the frequency-domain analysis.

Finally, for the temperature signal, visual inspection showed that the recorded signals did not contain significant artifacts, therefore no pre-processing was needed.

Further details concerning feature extraction are described in our previous works (Greco et al., 2016; Posada-Quintero et al., 2016; Garzón-Rey et al., 2017; Arza et al., 2018) and Table 1 summarises the final list of features used in the analysis.

**TABLE 1:** List of extracted features from physiological signals.

Physiological signal	Extracted features	Definition
Electrocardiogram (ECG) or Photoplethysmography (PPG)	SDNN (bpm)	Standard deviation of intervals between beats considered normal (NN)
	RMSSD (bpm)	Root mean square of successive differences between adjacent NN intervals
	mHR (bpm)	Mean heart rate
	PHF (s^−2^)	HRV High frequency band power (0.15–0.4 Hz)
	PHFex (s^−2^)	HRV Power in extended high frequency band (0.15 Hz-mHR/2)
Pulse Arrival Time (PAT)	mPAT (ms)	Mean pulse arrival time
	stdPAT (ms)	Standard deviation of the pulse arrival time
Electrodermal Activity (EDA)	mTonic (μS)	Average value of the tonic component, i.e. Skin Conductance Level (SCL)
	stdTonic (μS)	Standard deviation of the tonic component
	mPhasic (μS)	Average value over time of the phasic component
	stdPhasic (μS)	Standard deviation of the phasic component
	maxPhasic (μS)	Maximum peak value of the curve of the phasic component
	aucPhasic (μS·s)	Area under the curve value of the phasic component normalised by the length of the session, i.e. Skin Conductance Responses (SCRs)
	EDASymp (µS^2^)	Power spectral density of EDA signal in the frequency range of 0.045–0.25 Hz
Skin Temperature (T)	mT (°C)	Mean temperature
	mGrad (°C)	Mean temperature gradient
	medGrad (°C)	Median temperature gradient

### 2.4 Statistical analysis

The results of the psychometric tests and biochemical variables were analysed using mixed models with the subject as a random factor. All analyses were run in SAS 9.4 software. To fulfil the assumption of normality required by the model, the Box-Cox transform with *λ* = 0 was used when it was necessary. Principal components analysis (PCA) was performed to find the so-called Stress Reference Scale weights, and the most relevant features extracted from the physiological signals. Afterwards, we used linear regression with these physiological features to generate the Acute Stress Model. Before performing the PCA analysis, the features to be included in the model were re-scaled to a range of 0–100 in order to avoid unfair weighting given the differences in the order of magnitude of the values of each feature.

## 3 Results

### 3.1 Participants

The study included 120 healthy young students, recruited from the University of Zaragoza, the Technical University of Madrid and the Autonomous University of Barcelona, Spain. After individual checking for criteria fulfilment, 40 candidates per site were able to participate in the two different sessions.

The population studied corresponds to a group of 60 men and 60 women. Their average age is 22 ± 3 years and in normal ranges according to the WHO based on their body mass index (BMI) (Barba et al., 2004). 10% of the sample subjects suffered from asthma, respiratory allergies, migraine or intestinal reflux, but they were treated and it did not represent an inconvenience to carry out the measurement sessions. Most of the subjects had extracurricular activities, mainly sports, language courses or some type of artistic activities.

The perceived stress measured prior to the inclusion of the study did not exceed 47 units on average where from a scale from 0 to 100 it would be considered from no to mild stress. The self-perception of stress in subjects who were not drug consumers was higher (VAS = 46.21) than in those who consumed at least once a day (VAS = 15.3) but lower than in those who consumed less than once a day on average (VAS = 52.08).

The consumption habits were mainly non-toxic in the measured sample. In other words, non-use of tobacco or drugs and occasional consumption of liquor prevailed. The consumption or not of coffee was evenly distributed between men and women. Regarding the health status of the subjects based on Table 2, it can be defined as good or healthy. Given that most practice sports regularly or occasionally, they do not have diagnosed degenerative diseases and the consumption of pharmaceutical drugs was limited to the use of oral contraceptives. Finally, the majority of the subjects had a partner or emotional relationship, lived in family homes or shared a flat which suggested that there was no anomaly in the capacity for social interaction. In conclusion, the 120 subjects who participated in the measurement sessions met the proposed inclusion/exclusion criteria.

**TABLE 2:** Sociodemographic data.

Variables	Categories	*N* = 120
Age (m, std)		21,992 (2,865)
BMI (m, std)		22,145 (2,760)
Gender (n,%)	Female	60 (50)
	Male	60 (50)
Smoker (n,%)	No	100 (83)
	Yes	20 (16)
Coffee (n,%)	No	59 (49)
	Yes	61 (50)
Alcohol (n,%)	None	22 (18)
	Occasional	88 (73)
	Moderate	10 (8)
Drugs (n,%)	No	104 (86)
	Occasional	13 (10)
	Habitual	3 (2)
Chronic diseases (n,%)	No	108 (90)
	Yes	12 (10)
Pharmaceutical drugs (n,%)	No	94 (78)
	Yes	26 (21)
Relationship (n,%)	No	33 (27)
	Yes	87 (72)
Origin (n,%)	Rural	22 (18)
	Urban	98 (81)
Cohabitation (n,%)	Alone	6 (5)
	Family	76 (63)
	Shared_flat	38 (31)
Sport (n,%)	No	21 (17)
	Occasional	40 (33)
	Habitual	59 (49)

### 3.2 Outcome measures

#### 3.2.1 Psychometrics tests

The psychometric scores were calculated following the guidelines available in the CIBER-SAM bank of instruments and methodologies in mental health. The following tables summarise the results obtained from the psychometric test administered: Table 3.

**TABLE 3:** Psychometric test results.

	Total		Female		Male	
	RS	SS	RS	SS	RS	SS
PSS	20.68±(2.91)	20.00±(3.25)	20.65±(3.16)	20.41±(3.28)	20.70±(2.67)	19.59±(3.21)
STAIt	19.93±(9.53)	19.52±(9.53)	20.83±(9.80)	19.71±(9.84)	19.02±(9.24)	19.33±(9.29)
STAIs	15.99±(8.41)*	23.45±(11.37)*	16.03±(8.75)*	25.05±(11.10)*	15.95±(8.13)*	21.83±(11.52)*
VAS	32.68±(21.36)*	51.02±(21.18)*	34.85±(23.58)*	52.56±(19.48)*	30.50±(18.84)*	49.45±(22.85)*
SSS	21.21±(13.39)*	28.84±(15.05)*	21.92±(14.41)*	30.73±(14.64)*	20.50±(12.37)*	26.91±(15.36)*

Data are Mean and Standard deviation. RS: Relax State. SS: Stress State. PSS: Perceived Stress Scale. STAIs: State Anxiety Inventory. STAIt: Trait Anxiety Inventory. SSS: Symptomatic Stress Scale. VAS: Visual Analog Scale. * Statistically significant differences between RS, and SS (*p* < .05).

The Perceived Stress Scale scores were calculated as the sum of all the items with the reverse scores for questions 4, 5, 7, and 8. The results for each of the sessions and by gender were not significant. The studied sample had approximately three more units in the total score (2.76 ± 3, *p*-value < .0001) than the normative data of the PSS reported (Remor, 2006). This indicates that the studied population was more likely to perceive a situation as stressful or was more sensitive to it. There was no statistical evidence that this feature changed from one session to the next or that it was influenced by gender.

For the STAI, scores were calculated as the sum of all the items with the reverse scores for questions 1, 2, 5, 8, 10, 11, 15, 16, 19, 20, 21, 23, 26, 27, 30, 33, 34, 36, and 39. In the case of the STAIt, there were no statistically significant changes observed, which is consistent with the result of the Perceived Stress Scale. On the other hand, there was an increase in the scores for STAIs in the stress session compared to the relaxation session, regardless of gender. This result supports that the stressful stimulus applied in the stress session had an effect on modifying the state of the subjects. Women had a significantly lower trait (*p*-value = .0009) than the score reported in (Guillen-Riquelme and Buela-Casal, 2011), but an equivalent score to men.

The scores of the Visual Analog Scale and the Symptomatic Stress Scale had statistically significant increases that were consistent with the State Anxiety Inventory. Again, this result provides evidence of the stressful effect of the stress session and the correct application of the stimulus. Likewise, there were no differences related to sex.

The Symptomatic Stress Scale (SSS) was validated with a sample of 71 individuals by JM Garzón and published in his PhD thesis (Garzón-Rey J.M., 2017). The score is the sum of all the items with no reverse scoring. Internal validation of the SSS scale was carried out as an instrument for measuring acute emotional stress through a descriptive and inferential statistical analysis following the guidelines and procedures described in (Cook et al., 2006). Significant differences in the means of the relaxation and stress sessions were observed through a *t*-test for repeated measures with a *p* < .0001 in all state tests and no difference in the trait tests as expected. Cronbach’s Alpha Coefficient was determined for each of the two sessions from the descriptive statistics for each of the questions on the scale. No negative correlations or Cronbach’s Alpha Coefficient less than 0.8 were found, that is, the scale has internal consistency or reliability in both measurement states for the entire set of questions.

#### 3.2.2 Biochemical variables

The differences between the second sample and first sample of cortisol and α-amylase concentrations are shown in Table 4. Concerning cortisol, there was a lower decrease in concentration in the stress session compared to the relax session, that is, in both cases, between the two measurements of each session, the circadian cycle is observed, a decrease in cortisol and an increase in α-amylase. However, the lower decrease in cortisol in the stress session shows a greater release of cortisol opposite to the variation due to the circadian cycle. And the same occurs with α-amylase, there is a greater increase in the stress session than in the relaxation session. Both results showed greater activity in the HPA axis and in the sympathetic system during stress. Moreover, men had a higher concentration of cortisol than women while no differences were found in α-amylase concentrations.

**TABLE 4:** Biochemical variable results.

	Total		Female		Male	
	RS	SS	RS	SS	RS	SS
ΔCortisol ^⚤^	−0.06±(0.09)*	−0.02±(0.11)*	−0.04±(0.07)*	−0.02±(0.08)*	−0.08±(0.11)*	−0.01±(0.13)*
Δα-Amylase	9.22±(56.48)*	49.71±(77.76)*	10.50±(50.09)*	46.00±(78.31)*	7.92±(62.73)*	53.48±(77.69)*
Copeptin ^⚤^	6.28±(3.64)*	7.28±(3.97)*	4.74±(2.71)*	5.67±(3.46)*	7.75±(3.81)*	8.88±(3.82)*
Glucose	87.14±(14.77)	88.22±(9.80)	85.36±(14.95)	87.03±(8.28)	88.87±(14.51)	89.39±(11.05)
Prolactin ^⚤^	7.95±(3.62)*	9.16±(4.54)*	8.92±(4.27)*	10.45±(4.93)*	7.02±(2.58)*	7.92±(3.77)*

*Data are Mean and Standard deviation*. RS: Relax State. SS: Stress State. * Statistically significant differences (*p* < 0.05).

^⚤^ Statistically significant differences between gender.

As expected, there was a statistically higher value for prolactin and copeptin in the stress session with respect to the relaxation session. However, glucose levels were not significantly altered by the stressful stimulus applied.

Likewise, it is noteworthy that the values of all the variables were within the normal range of clinical use. It was also observed that the values were affected by sex, finding a higher concentration of copeptin in men than in women. On the contrary, the prolactin concentration in women was higher than in men. In fact, the concentration of prolactin in men in the stress session is equivalent to that of women in the relaxation session.

#### 3.2.3 Physiological signals

The results on the physiological features extracted during each protocol stage are presented on Table 5.

**TABLE 5:** Physiological features.

Electrocardiogram				
—	—	Mean	Std	No Diff
mHR [BMP]	BR	73,6	11,55	BS SA VD
	R	72,85	11,22	BS VD
	BS	72,99	11,85	BR R VD
	ST	82,81	15,84	MT
	MT	83,74	16,41	ST
	SA	73,85	12,92	BR VD
	VD	73,22	12,84	BR R BS SA
	AT	89,74	17,71	—
SDNN [s^−2^]	BR	0,13	1,2	R BS ST AT
	R	0,11	0,87	BR BS ST
	BS	0,07	0,08	BR R ST AT
	ST	0,22	1,01	BR R BS SA VD AT
	MT	0,35	1,62	
	SA	0,31	6,38	ST VD AT
	VD	0,59	3,85	ST SA AT
	AT	0,18	0,8	BR BS ST SA VD
RMS [s^−2^]	BR	0,11	1,71	R BS ST
	R	0,07	1,24	BR BS ST
	BS	0,03	0,11	BR R ST
	ST	0,25	1,42	BR R BS SA AT
	MT	0,38	2,26	VD
	SA	0,36	9,05	ST AT
	VD	0,77	5,45	MT
	AT	0,19	1,16	ST SA
PLF [s^−2^]	BR	0,61	0,81	ST SA
	R	0,51	1,22	BS ST
	BS	0,5	0,85	R ST SA
	ST	0,83	4,13	BR R BS SA
	MT	1,22	2,43	AT
	SA	0,5	0,61	BR BS ST
	VD	0,68	2,5	
	AT	0,86	2,24	MT
PHF [s^−2^]	BR	0,24	0,27	R BS ST SA VD
	R	0,23	0,25	BR ST SA VD
	BS	0,28	0,42	BR ST SA VD
	ST	0,57	3,34	BR R BS SA VD
	MT	0,75	2,02	
	SA	0,26	0,35	BR R BS ST VD
	VD	0,34	0,82	BR R BS ST SA
	AT	0,48	0,94	
PHFex [s^−2^]	BR	0,27	0,29	R BS ST SA VD
	R	0,26	0,38	BR ST VD
	BS	0,31	0,46	BR ST SA VD
	ST	0,62	3,41	BR R BS SA VD
	MT	0,87	2,26	
	SA	0,3	0,41	BR BS ST VD
	VD	0,38	0,9	BR R BS ST SA
	AT	0,58	1,18	
LF_HF [DL]	BR	380,56	489,64	ST MT SA AT
	R	299,32	423,79	ST MT SA
	BS	297,94	391,1	VD
	ST	284,94	253,19	BR R MT SA AT
	MT	284,73	382,89	BR R H SA AT
	SA	295,95	352,11	BR R ST MT AT
	VD	209,97	310,99	BS
	AT	311,26	492,72	BR ST MT SA

Photoplethysmography				
mHR [BPM]	BR	73,68	11,36	BS SA VD
	R	73,08	12,93	BS SA VD
	BS	73,37	11,74	BR R SA VD
	ST	83,12	16,72	MT
	MT	85,4	17,27	H
	SA	72,04	12,43	BR R BS VD
	VD	72,78	12,55	BR R BS SA
	AT	88,68	19,17	—
SDNN [BPM]	BR	0,02	0,01	R BS H
	R	0,02	0,01	BR BS H
	BS	0,02	0,01	BR R H
	ST	0,02	0,01	BR R BS VD AT
	MT	0,02	0,02	—
	SA	0,08	1,3	—
	VD	0,02	0,02	ST AT
	AT	0,02	0,01	ST VD
RMS [BPM]	BR	1,23	0,19	BS SA VD
	R	1,22	0,22	BS SA VD
	BS	1,23	0,2	BR R SA VD
	ST	1,39	0,28	MT
	MT	1,43	0,29	H
	SA	1,21	0,21	BR R BS VD
	VD	1,22	0,21	BR R BS SA
	AT	1,48	0,32	—

Electrodermal Activity				
mTonic [DL]	BR	−0,18	0,68	R
	R	−0,26	0,69	BR
	BS	−1,09	0,57	—
	ST	0,10	0,55	SA MT VD
	MT	0,12	0,62	SA ST VD
	SA	−0,03	0,52	ST MT
	VD	0,18	0,58	ST MT
	AT	0,40	0,79	—
stdTonic [DL]	BR	0,11	0,12	BS
	R	0,15	0,19	ST
	BS	0,10	0,16	BR
	ST	0,16	0,15	S MT R AT VD
	MT	0,20	0,22	SA ST AT VD
	SA	0,19	0,16	ST MT VD
	VD	0,18	0,19	SA ST MT AT
	AT	0,22	0,23	ST MT VD
EDASymp [DL]	BR	0,38	1,17	BS R
	R	0,90	3,05	BR BS
	BS	0,44	4,66	BR R
	ST	5,96	50,54	MT AT
	MT	1,73	4,01	ST AT
	SA	1,08	3,63	—
	VD	1,65	3,80	—
	AT	1,85	4,94	ST MT
mPhasic [DL]	BR	0,07	0,14	BS
	R	0,10	0,19	BS
	BS	0,07	0,17	BR R
	ST	0,38	0,45	SA MT AT VD
	MT	0,39	0,39	ST AT VD
	SA	0,22	0,25	ST VD
	VD	0,28	0,30	SA ST MT AT
	AT	0,35	0,35	ST MT VD
stdPhasic [DL]	BR	0,07	0,12	BS
	R	0,10	0,16	—
	BS	0,06	0,14	BR
	ST	0,17	0,28	MT AT VD
	MT	0,21	0,23	ST AT VD
	SA	0,17	0,17	—
	VD	0,19	0,20	ST MT AT
	AT	0,19	0,21	ST MT VD
maxPhasic [DL]	BR	0,29	0,48	BS R
	R	0,42	0,66	BR BS
	BS	0,25	0,51	BR R
	ST	0,82	1,03	MT AT
	MT	0,93	0,88	ST AT
	SA	0,70	0,68	—
	VD	0,80	0,76	—
	AT	0,88	0,86	ST MT
aucPhasic [DL]	BR	4,10	8,05	BS MT R AT SA VD
	R	5,99	11,31	SA BR BS MT AT VD
	BS	4,08	9,96	SA BR MT R AT VD
	ST	21,84	25,49	
	MT	22,67	23,15	SA BR BS R AR VD
	SA	12,88	14,15	BR BS MT R AT VD
	VD	16,59	17,49	SA BR BS MT R AT
	AT	20,36	20,73	SA BR BS MT R VD

Skin Temperature				
MeanT [°C]	BR	33,71	3,33	—
	R	34,7	2,83	H
	BS	34,31	3,28	H
	H	34,03	2,63	R BS
	MT	32,5	2,74	SA
	SA	32,3	3,49	MT
	VD	31,68	3,69	—
	AT	30,79	3,52	—
meanGrad [°C]	BR	0,03	0,07	BS
	R	0	0,04	SA
	BS	0,03	0,07	BR
	ST	−0,07	0,07	MT
	MT	−0,07	0,07	H
	SA	0	0,08	R
	VD	−0,04	0,06	AT
	AT	−0,04	0,06	VD
medGrad [°C]	BR	0,03	0,07	BS
	R	0	0,04	SA
	BS	0,03	0,07	BR
	ST	−0,07	0,07	MT
	MT	−0,07	0,07	H
	SA	0	0,09	R
	VD	−0,04	0,07	AT
	AT	−0,04	0,06	VD

Pulse Arrival Time				
mPAT [ms]	BR	281,69	69,46	R BS ST MT SA
	R	277,41	65,41	BR BS ST MT SA
	BS	270,06	66,77	BR R ST MT SA VD AT
	ST	259,48	77,5	BR R BS MT SA VD AT
	MT	277,9	90,85	BR R BS ST SA VD AT
	SA	268,59	69,31	BR R BS ST MT VD AT
	VD	256,59	68,02	BS ST MT SA AT
	AT	258,3	88,29	BS ST MT SA VD
stdPAT [ms]	BR	15,12	29,98	R BS ST SA VD
	R	13,69	29,43	BR BS ST SA VD
	BS	14,64	39,88	BR R ST SA VD
	ST	19,88	52,31	BR R BS MT SA AT
	MT	30,11	49,36	ST AT
	SA	18,41	56,6	BR R BS H
	VD	12,76	40,03	BR R BS
	AT	22,66	42,88	ST MT

EDA values multiplied by 100. DL: Dimensionless. BR: Basal relax. R: Relax. BS: Basal stress. ST: Storytelling. MT: Memory test. SA: Stress Anticipation. VD:Public video display. AT: Arithmetic Task. No Diff: No significant difference with the stage listed (*p*-value < .05).

For the HRV, regarding the temporal indices, none of them differed between the basal moments but they were significantly different compared to the AT stage, which is the moment of the highest stress level. However, it is surprising that the moments of relaxation, VD and SA did not differ. The frequency indices in both the classic and the extended bands differed between the two moments BR and BS, failing to meet one of the selection criteria. The only frequency indices that met this criterion were the indices in the HF band, although this was only calculated when the subject was quiet. In conclusion, the heart rate variability features that were considered adequate for estimating the stress level were: the mean value (mHR), the standard deviation (SDNN) and the mean square value of the heart rate (RMS) and in those cases where the subjects were not speaking, the indices on the HF band. Similar results were found in the PRV analysis.

When combining both signals, no significant differences were found between the two baselines BR and BS for both mPAT and stdPAT. In the stages where the stressful stimulus was applied, a decrease in pulse arrival time and greater variability were observed.

All the features from the electrodermal activity except the skin conductance level met the selection criteria of being the same at the baseline moments of both sessions and showed a significant difference between the moments of relaxation and the moments in which the stressful stimulus was applied. It is important to highlight that, for the EDA analysis, 40 subjects of the total sample of 120 were discarded due to configuration problems with the measurement equipment, therefore the results presented correspond to only 80 subjects.

Finally, for the skin temperature features, only the temperature gradient in the finger (mGrad and medGrad) met the selection criteria since there were no differences at the two baseline moments (BS and RB). A temperature decrease was observed during moments of stress, which was more intense in stages ST and MT than VD and AT.

### 3.3 Stress Reference Scale

Following the methodology suggested in (Garzon-Rey et al., 2017), a Stress Reference Scale (SRS) was computed as a weighted average of the psychometric test scores and biochemical variables. The weights for each element were assigned in accordance with a principal components analysis (PCA) being proportional to the scoring coefficient and the variances of the component. To define the SRS, the components with an eigenvalue higher than 0.9 that together explained at least 75% of the total variance were selected.

As shown in Table 6, the first five components fulfilled the selection criteria of an eigenvalue higher than 0.9 and accumulated explained variance at least 75% of the total variance. The resulting scoring coefficients for the five components are shown in Table 7 and the highest values are highlighted. The first and the fourth component had the highest scores for the psychometric tests. Components 2 and 3 can be associated with the HPA activation since the cortisol, copeptin and prolactin scores are the most relevant among these components. Finally, the last component, the fifth, has the peak value for the α-amylase that gives this component a relation to the sympathetic nervous system.

**TABLE 6:** Eigenvalues and explained variances for components of PCA.

Comp	Eigenvalue	Dif	% VAR	VAR Acum (%)
**1**	2.78	1.56	31	31
**2**	1.22	0.12	14	44
**3**	1.10	0.12	12	57
**4**	0.99	0.04	11	68
**5**	0.95	0.16	11	78
6	0.78	0.20	9	87
7	0.58	0.22	6	93
8	0.36	0.11	4	97
9	0.24		3	100

Comp: component. % VAR: Percentage of explained variance. VAR, Acum: Percentage of accumulated explained variance.

**TABLE 7:** Scoring coefficients for computing component scores.

Component	1	2	3	4	5
STAIs	**90**	10	−1	0	4
SSS	**87**	2	13	1	-4
VAS	**79**	12	−13	−3	23
STAIt	**69**	-6	5	31	-20
PSS	9	0	−11	**93**	5
Cortisol	5	**77**	29	20	0
α-Amylase	3	3	3	4	**97**
Copeptin	3	1	**92**	−11	3
Prolactin	7	**75**	−27	−21	5
% VAR	40%	17%	16%	14%	13%

VAR, Prop: Percentage of Total Variance Explained by Component.

Bold text indicates values >60.

The resulted Stress Reference Scale (SRS) computed for a population of 120 young people was:

SRS = 0.14*PSS + .11*SSS + 0.11*STAIs + .10*VAS + 0.08*STAIt + .16*Copeptin + .13*⍺Amylase + .09*Cortisol +0.08*Prolactin.

Table 8 shows that there were no significant differences between male and female SRS scores, although, as expected, the SRS was significantly higher in the Stress Session compared to the relax session.

**TABLE 8:** Stress Reference Scale results.

SRS		N	Mean	Std	Min	Q1	Median	Q3	Max
Session*	RS	114	39,40	6,70	27,28	35,09	38,01	42,70	62,34
	SS	113	45,40	7,78	29,81	40,26	45,34	51,01	71,31
Gender	F	112	42,89	7,89	28,59	37,56	41,08	47,30	71,31
	M	115	41,90	7,80	27,28	36,40	40,43	47,94	60,13

*p < .05.

### 3.4 Physiological stress model

To generate the acute stress model, first of all, feature values that were significantly different at the baseline stages of both sessions were discarded considering that their values were reflecting conditions other than the stress. Moreover, features affected by speech such as respiratory rate were also excluded to avoid intrinsic noise.

Unlike the Stress Reference Scale, where values can be associated only with a state of stress (SS) or a state of relaxation (RS), the physiological features selected are, in a way, “continuous.” Although, due to their method of calculation, they are associated with different moments of the session, and, therefore, offer information on the different intensities of stress reached at this time.

To select the relevant features, we associated the values of the SRS for the state RS to the features calculated at the moment of R of the first session measurement, and the values of the SRS in the SS state to the last moment of the second measurement session, that is, to the AT stage.

Two sequential selections were done in order to simplify further analysis. Firstly, from each set of features showing statistically significant linear dependencies, only one was selected. From this set of features, only those having a significant correlation with the SRS score were selected in a second step. Only 18 out of the initial 80 physiological features were finally selected: from the ECG the HRV_SDNN, HRV_RMS, HRV_PHFex, HRV_PHF and HRV_mHR; from the PPG the PRV_SDNN, PRV_RMS and PRV_mHR, from the PAT the mPAT and stdPAT; from the EDA the stdTonic, stdPhasic, maxPhasic, mPhasic, aucPhasic and EDASymp; and from the ST the mGrad and medGrad. It was considered that this number of features could be easily handled to give results in real-time without requiring large computing capabilities.

Table 9 shows the eigenvalues and explained variances for the components resulting from the PCA analysis on the set of features. It is observed that the first five components are the only ones that have an eigenvalue greater than one and with them, 87% of the variance of the data is explained, which is sufficient for the type of analysis that was carried out.

**TABLE 9:** Eigenvalues and explained variances for components of PCA.

Comp	Eigenvalue	Dif	% VAR	VAR Acum
1	5.79	1.27	32	32
2	4.52	2.35	25	57
3	2.17	0.27	12	69
4	1.90	0.54	11	80
5	1.36	0.72	8	87
6	0.63	0.17	4	91
7	0.46	0.03	3	93
8	0.43	0.11	2	96
9	0.31	0.13	2	98
10	0.19	0.06	1	99
11	0.12	0.08	1	99
12	0.04	0.01	0	100

Comp: component. % VAR: Percentage of explained variance. VAR, Acum: Percentage of accumulated explained variance.

For the five components selected, Table 10 shows the coefficients or associated weights of each feature. It is observed that each feature loads in a single component with quite a difference compared to the others. This fact can be understood as a great collinearity between the features that make up each group and therefore an evident redundancy in the information they offer. To select the features included for each of the components, only those that had a coefficient greater than 60 were selected.

**TABLE 10:** Scoring coefficients for computing component scores.

	C1	C2	C3	C4	C5
HRV_mHR	21	−7	**91**	−4	15
HRV_SDNN	11	**77**	−40	0	6
HRV_RMS	1	**79**	−34	−1	3
HRV_PHF	3	**94**	18	0	0
HRV_PHFex	5	**94**	21	−1	2
stdTonic	**79**	3	1	−5	5
mPhasic	**98**	5	9	−5	1
stdPhasic	**98**	2	7	−3	1
maxPhasic	**98**	2	8	−4	1
aucPhasic	**98**	5	9	−5	1
EDASymp	**94**	7	10	−7	3
PRV_mHR	19	−6	**93**	−3	14
PRV_SDNN	9	**65**	−56	4	7
PRV_RMS	0	**65**	−57	1	0
mPAT	−2	−10	7	1	**90**
stdPAT	10	21	14	−4	**86**
meanGrad	−10	0	−3	**99**	−1
medGrad	−9	1	−4	**99**	−2

VAR, Prop: Percentage of Total Variance Explained by Component.

Bold text indicates values >60.

The first component was associated with all the features extracted from the electrodermal activity, which in turn is associated with the state of the sympathetic system. The phasic component from the EDA signal revealed to provide greater information among the EDA parameters dimension. We selected the standard deviation of the phasic component because it showed better correlation with the SRS.

The group of features associated with the second component was composed of the heart rate and pulse rate variability indices related to the power in the HF band. The features HRV_PHFex and HRV_PHF had the highest coefficients associated with this component, but HRV_PHFex was selected since its weight was slightly higher.

The mean values of heart rate estimated from the ECG and PPG were in the third component group. The differences in weight between the two features were very small and although the weight corresponding to the one coming from the PPG (PRV_mHR) was strictly greater, in this case, other reasons were considered. On the one hand, taking the HRV_mHR feature (from the ECG) would avoid having to deal with the greater number of artefacts that PPG undoubtedly generates and, in turn, would eliminate the need to take the PPG signal into consideration since the previous group already took the ECG. On the other hand, using the PPG represents greater comfort for the subject, since the signal can be extracted from a watch or bracelet and adhered electrodes would not be necessary. However, this option makes no sense if we keep the choice of HRV_PHFex in the previous group (which already requires having the ECG). On the contrary, selecting PPG in both groups would definitely mean greater comfort for the subject at the cost of losing precision in the second component.

The mean and median of the hand temperature gradient were the features that form the fourth group. The coefficient associated with the fourth component was identical for both features. mGrad was selected since calculating the mean is computationally less expensive than the median.

Finally, the last group, associated with the fifth component, corresponded to the pulse arrival time features. Among these, we selected the mean value (mPAT) for having the highest coefficient associated with component five. From the selected features and using a linear regression model, the Stress Reference Scale was estimated, which resulted in the following equation with a determination coefficient R2 of 0.97:

PSM = .5 · mGrad + .4 · PRV_mHR + .2 · HRV_PHFex + .06 · mPAT + .01 · stdPhasic.

Table 11 shows the descriptive statistics of the physiological stress model. A net difference between the stress states SS and relaxation states RS is preserved.

**TABLE 11:** Physiological Stress Model results.

SRS		N	Mean	Std	Min	Q1	Median	Q3	Max
Session*	RS	114	40.48	3.62	26.84	38.45	40.7	42.54	52.98
	SS	113	44.64	4.74	30.71	41.87	44.55	48.03	52.83
Gender	F	112	41.35	4.01	30.71	38.69	41.18	43.56	52.83
	M	115	40.87	4.14	26.84	38.81	40.83	43.18	52.98

*p < .05.

Similar to the SRS results, there were no significant differences between male and female PSM scores, although, as expected, they were significantly higher (*p* < 0.05) in the Stress Session compared to the relax session.

## 4 Discussion

Stress is a phenomenon that we can perceive, but hardly quantified. The aim of this study was to establish a quantitative, continuous, objective and non-invasive measure of the level of acute emotional stress in healthy young subjects. From a practical point of view, this tool will help clinicians improve a more accurate diagnosis and during the follow-up of therapeutic interventions while facilitating a common ground among professionals.

### 4.1 Artificial stress environment protocol

First of all, from the literature, we have seen that there have coexisted three distinct approaches to the stress assessment. The first one relies on the subject’s lifetime stressful experiences, which generate a higher affectation level, the second one evaluates the psychological perception of a stressful stimulus and the third one measures some variables of the physiological response of the subject (Hellhammer et al., 2010). Gathering information from these three approaches, we have designed a protocol that includes two different sessions. The relax session in which a state of relaxation is achieved with music. The stress session begins with a brief relaxation time to reach similar conditions to the previous session. The stress state is then achieved using the TSST as a moderate stressor. At the end of both sessions, various psychometric tests and biochemical markers are administered as accepted gold standards for stress assessment in humans and animal models respectively. The psychometric tests evaluate the psychological affectation due to the sequence of events while the biochemical variables evaluate the response of the endocrine system. Additionally, several electrophysiological variables, reported in the literature as stress markers, are also measured synchronously throughout the sessions to assess ANS activation.

The Trier Social Stress Test is an experimental protocol that has made it possible to study the relationship between psychological and physiological responses in recent decades (Kudielka, B.M. et al., 2007). Its usefulness lies in sequentially including various different common types of stressors: a memory task, a social interaction event and an arithmetic task, all without previous warning.

The reasoning behind the two separate sessions in the design of the present study considered the effect that could resemble the commonly known as “white coat syndrome”, in which participants are altered due to the clinical or experimental setting, although they do not exhibit it in other settings. On the one hand, participants knew what to expect during the second session regarding the sensor placement and the experimental setting and on the other hand, the features that were significantly different in both basal states (BR and BS) were discarded considering their variations were not directly related to stress. Furthermore, this protocol allowed all subjects to be their own control reducing possible biases.

### 4.2 Reference scale for stress measurement

Once the experimental setting was designed, our initial scope was to find a comprehensive scale for stress measurement that could be later used as reference for more practical use cases offering synthesised information on both the response corresponding to the «cogno » or of the psyche as well as of the somatic response to a stressful stimulus.

To that end, there are two sets of highly correlated systems that activate simultaneously when faced with a stressful stimulus, generating a single response. The first is associated with changes in the psyche or « cogno», i.e. changes in thinking, the generation of emotions, the perception of reality, behaviour and social interaction, among others. The second set is associated with somatic changes from the system or organ level to the molecular level. This in turn is activated by two separate although interrelated and cooperative pathways: the hypothalamic-pituitary-adrenal (HPA) axis and the autonomic nervous system (ANS).

From the cognitive point of view, our study results clearly showed the stress reactivity caused by the artificial stressor; the questionnaires evaluating the imminent stressful situation, i.e., STAIs, VAS and SSS, showed at least a 10% increase in the stress session, whereas the trait questionnaires, i.e., STAIt and PSS, remained the same. These results are in accordance with the reported literature (Kudielka, et al., 2007; Bae et al., 2018).

Regarding the HPA axis, results show a lower decrease in cortisol concentration during the stress session compared to the relax session. Previously documented patterns of higher anticipatory salivary cortisol responses during the TSST in men compared to women have also been found (Liu et al., 2017). Cortisol release also increases the glucose availability for a fight or flight response although our results do not show significant differences.

The synthesis and release of cortisol in humans are subject to a circadian rhythm, with high levels in the early morning that decrease as the day goes by. Additionally, the secretion of cortisol is stimulated as a result of the activation of the HPA axis triggered by a stressful stimulus. Even though the morning cortisol may be difficult to compare during the cortisol awakening response, stress reactivity in healthy subjects has been proven to show greater response compared to the evening results (Yamanaka et al., 2019).

Outside the adrenal gland, cortisol is mainly bound to corticosteroid-binding globulin (CBG). The remaining part, called free cortisol, represents the biologically active part of the hormone. The cortisol found in saliva corresponds to the free cortisol that diffuses passively into the salivary glands and its concentration is associated with the levels of free cortisol in plasma (Foley and Kirschbaum, 2010). Although salivary cortisol can be altered as a consequence of a variation in the CBG concentration (Hellhammer et al., 2009), we analysed it since it still has numerous advantages over its measurement in plasma, as it does not require any equipment or specialised personnel for its collection.

The activation of the HPA axis also gives rise to arginine vasopressin (AVP) and prolactin hormones secretion. AVP has a small molecular size and a short average lifetime that makes it difficult to measure. Therefore, we determined the concentration of a co-secreted glycopeptide, known as copeptin, as a surrogate biomarker, although physiological interpretation is unknown (Carrillo-Esper et al., 2013; Urwyler et al., 2015). Both prolactin and copeptin blood concentrations were elevated after the stressor.

Regarding the ANS, the sympathetic activation of the salivary glands increases the secretion of α-amylase while that of the parasympathetic increases the volume of secreted saliva (Garrett et al., 1999). Furthermore, the concentration of α-amylase also changes depending on the circadian rhythm of the subject inversely to cortisol (Rohleder et al., 2004).

The greater increase in the stress session compared to the relax shows the effect of the TSST, which has been associated with increased plasma catecholamines (adrenaline, noradrenaline) due to the presence of stressful stimuli. Therefore, there is a presumption that α-amylase in saliva could be considered a non-invasive substitute for the measurement of catecholamines and even cortisol (Rohleder et al., 2004).

To elaborate the Stress Reference Scale, all the above variables that respond to the activity of the hypothalamic-pituitary-adrenal axis and the sympathetic nervous system that significantly changed as a result of the TSST were considered in the principal component analysis and weighted according to their relevance. Each coefficient of each variable is proportional to the scores in the component and explained variances of the component. The inclusion of the trait psychometric test (i.e. PSS and STAIt), allowed the Scale to have the information of predisposition (trait) of the individual to respond to a stressful stimulus.

The SRS scale shows that 54% of the score corresponds to Psychometric tests, rather than 60% proposed in (Garzon-Rey et al., 2017). The difference can be explained due to the inclusion of the Symptomatic Stress Scale (SSS) test and α-amylase since the increase up to 120 participants showed significant concentration differences between Relax and Stress states, unlike the subset of 40 participants used in (Garzon-Rey et al., 2017). The SRS was validated for the type of population and stimulus of the present study and is independent of gender.

Once validated, the proposed SRS represented a quantitative assessment of stress on a scale from 0 to 100 in which SRS = 0 means the total absence of stress (no response to stimuli) and SRS = 100 means the highest level of distress.

### 4.3 Non-invasive stress measurement

Finally, our protocol included the synchronous monitoring of several non-invasive stress biomarkers. We extracted several stress-related physiological features found in the literature to study their evolution under different stress intensities and to find the most relevant ones for a continuous acute stress measurement.

In (Arza et al., 2018) an approach to estimate stress level every 1 min from physiological signals was evaluated on a subset of the database. Results supported the feasibility of quantifying the stress level using a multiparametric measure of the physiological stress response through biomarkers derived from the processing of ECG, PPG and ST signals. Moreover, the relationship between the features extracted from the signals and the SRS showed higher correlations than with either the psychometric variables or the biochemical variables alone. This suggested the feasibility of the SRS used to assess the stress levels.

In the present study, the conditioning and treatment of physiological signals was performed on ECG, PPG and ST, and EDA was also added. From them, more than 80 features widely reported in the literature or that are associated with physiological processes related to stress were extracted.

The heart rate variability features that were considered adequate for estimating the stress level were the mHR, the SDNN, RMS and HF band, in line with previous analysis in the data subset (Arza et al., 2015) where they significantly changed between highest stress stages (MT and AT) and all other stages of the session.

Regarding pulse rate variability (PRV), in (Garzón-Rey et al., 2017) the complementary or additional information of PRV with respect to HRV was investigated for stress assessment. While significant differences were found between PRV and HRV at baseline, no such differences were observed during stress stages, suggesting that pulse arrival time variability is higher during relax than during stress. Changes in the morphology of the PPG signal induced by stress were studied using a Gaussian mixture modelling technique, revealing significant differences in features related to the position and width of the systolic and reflected waves during stress stages with respect to baseline (Banerjee et al., 2017).

For the joint analysis of the ECG and PPG signals, we extracted the PAT features which reduced during the stressor. These are reported to be a proxy for vasoconstriction and hence higher blood pressure.

The PAT is the time difference between the peak of the R-wave in the ECG signal (corresponding to left ventricular depolarisation) and a fiducial point in the PPG waveform (as measured by a pulse oximeter attached to the fingertip). However, it is often erroneously described in literature as pulse transit time (PTT) (Finnegan et al., 2021). The PAT includes not only PTT, but also the time delay between the electrical depolarisation of the heart’s left ventricle and the opening of the aortic valve, known as pre-ejection period (PEP). PEP can vary depending on contractility, sympathetic nervous system activity, preload and afterload. In our study, we did not separate the PEP from the analysis, although the literature suggests that the time elapsed between the R-peak and the R-wave gives the best systolic blood pressure prediction (Wong et al., 2011).

For the skin temperature features, the experiments reported in the literature do not converge to the same conclusion. In this study, the mean temperature decreases rapidly during the first stages of the TSST, remains at its lowest values until the end of the whole task and it has been reported that more than an hour is needed post-stress for temperature to return to baseline (Pisanski et al., 2018).

Finally, electrodermal activity features measure a person’s sweat level in glands since the skin is usually an insulator but its conductance changes when there is sweat as a consequence of sympathetic activation. Skin conductance has been found to have a linearly varying property with respect to emotional arousal, and apart from being used to classify different states such as anger and fear, it is also to detect the stress level while performing a task (Baig et al., 2019). The EDA can be divided in two independent components; the first in which rapid, immediate responses are observed and of short duration, known as phasic component, and the second, the tonic component, is associated with the level of arousal of the sympathetic nervous system, that is, a sustained or accumulated response. Our results show this arousal in both components of the time-domain analysis and in the frequency-domain analysis, supporting the sympathetic arousal.

From the PCA analysis, the selected final set of five features explaining 87% of the variance of the data were:

PSM = .5 · mGrad + .4 · PRV_mHR + .2 · HRV_PHFex + .06 · mPAT + .01 · stdPhasic.

This model takes into account all the before mentioned features emphasising changes in the increase of the mean heart rate and the temperature decrease. Contrary to what the literature suggests, the power in the extended high frequency band of the HRV analysis increases with stress, which is usually related to parasympathetic activity (as reviewed by Kim et al., 2018). This could be explained because the power of the extended band of the HF goes from 0.15 Hz to half the heart rate. Therefore, if the heart rate increases during the stress stage, the band is extended and consequently the power can increase. Moreover, speech should be taken into account to increase the reliability of HRV as a marker of stress as frequency features of HRV are influenced by respiration (Hernando et al., 2016). During the relax state, respiratory frequency is within the LF band, so the HF band is not measuring respiratory sinus arrhythmia but just “noise”. On the contrary, during the stress tasks, respiratory frequency increases and falls within the HF band, thus increasing the power in the HF band. However, this can be misinterpreted as an increase in parasympathetic activity. An approach to better estimate the sympathovagal balance after separating respiratory influences from the heart rate was developed in (Varon et al., 2019). Finally, a minor contribution goes to the mean pulse arrival time and the standard deviation of the phasic component of the electrodermal activity.

Our previous publication on a subset of the database (Arza et al., 2018) where we designed a similar model for the non-invasive measurement of stress, the set of extracted features was more limited and the rescaling of the magnitudes to balance weightings given the differences in the order of magnitude of the values of each feature was not carried out. However, both models are able to quantitatively measure the stress changes over time and show a good correlation with the reference scale designed showing that the methodology used is reliable and can be adapted to the conditions needed for a specific end-user application. Moreover, after all the analysis, both models include the mHR and mPAT. Therefore, these features show robustness and should be considered for future stress assessment methods.

The purpose of this analysis is to achieve a continuous, non-invasive measurement that allows quantitatively assessing different levels of stress in a repeatable manner and from an approach that groups the different aspects of the psychosomatic changes induced due to a stressor. In fact, the Physiological Stress Model can be calculated throughout the entire session. The highest values of the model can be observed in MT and AT and the lowest in BR, R BS, as expected. It is important to highlight that due to the type of re-scaling that was carried out to “uniform” the ranges of the features, this model will never reach a minimum of 0 or a maximum of 100, which is adequate for the type of moderate stress in healthy students being studied.

### 4.4 Limitations

The main limitation of the current study is the specific target subject of study, narrowed to healthy young students and a perfect acute stress experimental setting although not applicable in a wider population and other types of stress. Further studies including clinical and sociodemographic data such as age, gender, BMI or fitness may enhance the model’s robustness.

The ultrashort term analysis for the HRV is a trade-off between usability and reliability and should also be more carefully studied. Moreover, respiration and speech inclusion into the analysis could greatly improve the reliability of the measurement.

Finally, only linear relations between the stress response and the stress reference features were analysed in this approach, although including non-linear relations could contribute to a more accurate measure of the stress reactivity nature.

## 5 Conclusion

The present study explores the multidimensionality of perceived stress *via* a carefully designed holistic protocol and analysis. To reach the proposed objectives a wide set of measurable variables related to the psychometric perception, hypothalamic-pituitary-adrenal axis and the autonomic nervous system response have been taken into account.

We used principal component analysis to find the variables that mostly contribute to differentiate the relax and stress states and weigh them according to the Stress Reference Scale. Therefore, the SRS gives the quantitative stress level of an individual based on the biochemical variables and the psychometric scores. The regression analysis with physiological variables to forecast the SRS level is then used to elaborate the Physiological Stress Model. Although the model has been developed to match the SRS values in the initial (relaxation) and final (stress) states, this model allows to observe changes in the level of stress throughout all the stages of the TSST continuously, non-invasively and specifically for acute emotional stress.

Additionally, the Symptomatic Stress Scale has been validated as a new psychometric test to assess the somatic and psycho-cognitive stress perceived symptoms. Sequeira et al., 2021.

## Data Availability

The raw data supporting the conclusion of this article will be made available by the authors, without undue reservation.

## Appendix A:

**TABLE A1:** Escala de síntomas de estrés (SSS).

Señale la opción que mejor describe cómo se siente en este momento		Muy de acuerdo	Algo de acuerdo	Ni de acuerdo ni en desacuerdo	Algo en desacuerdo	Muy en desacuerdo
1	Siento palpitaciones	—	—	—	—	—
2	Siento la boca seca	—	—	—	—	—
3	Siento rigidez en el cuello	—	—	—	—	—
4	Siento que me falta el aire, suspiro con frecuencia	—	—	—	—	—
5	Siento opresi n en el pecho	—	—	—	—	—
6	Siento escalofr os	—	—	—	—	—
7	Siento urgencia para orinar con frecuencia	—	—	—	—	—
8	Siento que sudo	—	—	—	—	—
9	Siento como si tuviera nervios en el est mago	—	—	—	—	—
10	Siento que mi cara se sonroja	—	—	—	—	—
11	Me siento mareado	—	—	—	—	—
12	Cometo muchos errores	—	—	—	—	—
13	No tengo ganas de hablar	—	—	—	—	—
14	Me siento enfadado	—	—	—	—	—
15	Me siento molesto por todo	—	—	—	—	—
16	Siento que me distraigo f cilmente o no me concentro	—	—	—	—	—
17	No me siento motivado para hacer cosas	—	—	—	—	—
18	Me siento al l mite	—	—	—	—	—
19	Siento que me impaciento f cilmente	—	—	—	—	—
20	Me siento agitado e/o inquieto	—	—	—	—	—

## References

[B1] Abend R. Dan O. Maoz K. Raz S. Bar-Haim Y. (2014). Reliability, validity and sensitivity of a computerized visual analog scale measuring state anxiety. Journal of Behavior Therapy and Experimental Psychiatry, 45 (4), 447–453. 10.1016/j.jbtep.2014.06.004 24978117

[B2] Aguilo J. Ferrer-Salvans P. Garcia-Rozo A. Armario A. Corbi A. Cambra F. J. (2015). Project ES3: Attempting to quantify and measure the level of stress. Revue neurologique, 61, 405–415. 10.33588/rn.6109.2015136 26503316

[B3] Allen A. P. Kennedy P. J. Cryan J. F. Dinan T. G. Clarke G. (2014). Biological and psychological markers of stress in humans: Focus on the trier social stress test. Neurosci. Biobehav. Rev 38, 94–124. 10.1016/j.neubiorev.2013.11.005 24239854

[B4] American Psychiatric Association (2013). Diagnostic and statistical manual of mental disorders. Fifth Edition. American Psychiatric Association. 10.1176/appi.books.9780890425596

[B5] Armario A. Marti O. Molina T. de Pablo J. Valdes M. (1996). Acute stress markers in humans: Response of plasma glucose, cortisol and prolactin to two examinations differing in the anxiety they provoke. Psychoneuroendocrinology 21 (1), 17–24. 10.1016/0306-4530(95)00048-8 8778900

[B6] Arza A. Garzón J. M. Hemando A. Aguiló J. Bailon R. (2015). Towards an objective measurement of emotional stress: Preliminary analysis based on heart rate variability. Annu. Int. Conf. IEEE Eng. Med. Biol. Soc 2015, 3331–3334. 10.1109/EMBC.2015.7319105 26737005

[B7] Arza A. Garzón-Rey J. M. Lázaro J. Gil E. Lopez-Anton R. de la Camara C. (2018). Measuring acute stress response through physiological signals: Towards a quantitative assessment of stress. Med. Biol. Eng. Comput 57, 271–287. 10.1007/s11517-018-1879-z 30094756

[B8] Bae Y. J. Reinelt J. Netto J. Uhlig M. Willenberg A. Ceglarek U. (2019). Salivary cortisone, as a biomarker for psychosocial stress, is associated with state anxiety and heart rate. Psychoneuroendocrinology 101, 35–41. 10.1016/j.psyneuen.2018.10.015 30408721

[B9] Baig M. Z. Kavakli M. (2019). A survey on psycho-physiological analysis & measurement methods in multimodal systems. Multimodal Technol. Interact 3 (2), 37. 10.3390/mti3020037

[B10] Bakker J. Holenderski L. Kocielnik R. Pechenizkiy M. Sidorova N. (2012). Stess@ work: From measuring stress to its understanding, prediction and handling with personalized coaching. Industrial Eng. Chem. Res. IND ENG CHEM RES 673-678. 10.1145/2110363.2110439

[B11] Banerjee S. Bailon R. Lazaro J. Marozas V. Laguna P. Gil E. (September 2017). A two step Gaussian modelling to assess PPG morphological variability induced by psychological stress. Proceedings of the 2017 Computing in Cardiology (CinC), IEEE, Rennes, France 10.22489/CinC.2017.270-035

[B12] Barba C. Cavalli-Sforza T. Cutter J. Darnton-Hill I. Deurenberg P. Deurenberg-Yap M. (2004). Appropriate body-mass index for Asian populations and its implications for policy and intervention strategies. Lancet 363, 157–163. 10.1016/S0140-6736(03)15268-3 14726171

[B13] Brämer G. R. (1988). International statistical classification of diseases and related health problems. Tenth revision. World Health Stat. Q 41, 32–36. 3376487

[B14] Carrillo-Esper R. Copeptina de la Torre-León T. (2013). U. N. novedoso interes. biomarcador pronóstico Int Mex 29, 380–387.

[B15] Cohen S. Kamarck T. Mermelstein R. (1983). A global measure of perceived stress. J. Health Soc. Behav 24, 385–396. 10.2307/2136404 6668417

[B16] Cohen S. Kessler R. C. Gordon L. U. (1997). Measuring stress: A guide for health and social scientists. A project of the Fetzer InstituteOxford University Press.

[B17] Cook D. A. Beckman T. J. (2006). Current concepts in validity and reliability for psychometric instruments: Theory and application. Am. J. Med 119 (2), 166.e7–16. PMID: 16443422. 10.1016/j.amjmed.2005.10.036 16443422

[B18] De Santos-Sierra A. Ávila C. S. Casanova J. G. Del Pozo G. B. (2011). Real-time stress detection by means of physiological signals. Recent Appl. Biometrics 58, 4857–4865.

[B19] Derogatis L. R. (1987). “The Derogatis stress profile (DSP): Quantification of psychological stress,” in Research paradigms in psychosomatic medicine, 17, 30–54. 10.1159/000414005 3591564

[B20] Finnegan E. Davidson S. Harford M. Jorge J. Watkinson P. Young D. (2021). Pulse arrival time as a surrogate of blood pressure. Sci. Rep 11 (1), 22767. 10.1038/s41598-021-01358-4 34815419 PMC8611024

[B21] Foley P. Kirschbaum C. (2010). Human hypothalamus–pituitary–adrenal axis responses to acute psychosocial stress in laboratory settings. Neurosci. Biobehav. Rev 35, 91–96. 10.1016/j.neubiorev.2010.01.010 20109491

[B22] Garrett J. R. John R. Ekström J. Anderson L. C. (1999). Neural mechanisms of salivary gland secretion. Karger, Basel, Switzerland,

[B23] Garzon-Rey J. M. Arza A. De-la-Camara C. Lobo A. Armario A. Aguilo J. (2017). An approach to an acute emotional stress reference scale. Rev. Neurol 64, 529–537. 10.33588/rn.6412.2016509 28608352

[B24] Garzon-Rey J. M. Arza A. Salama A. A. K. Caja G. Aguilo J. (April 2016). Environmental temperature changes as stress stimulus, Proceedings of the Global medical engineering physics exchanges/Pan American health care exchanges (GMEPE/PAHCE). Madrid, Spain, IEEE, 1–4. 10.1109/GMEPE-PAHCE.2016.7504666

[B25] Garzón-Rey J. M. Lázaro J. Milagro J. Gil E. Aguiló J. Bailón R. (2017). “Respiration-guided analysis of pulse and heart rate variabilities for acute emotional stress assessment, in Proceedings of the 2017 Computing in cardiology. IEEE, Rennes, France, 10.22489/CinC.2017.318-093

[B26] Garzón-Rey J. M. (2017). Psychosomatic approach to stress measurement [PhD thesis. Barcelona: Autonomous Univerity of Barcelona. Edith Cowan Online RepositoryRetrieved from https://hdl.handle.net/10803/458661.

[B27] Gil E. Mendez M. Vergara J. M. Cerutti S. Bianchi A. M. Laguna P. (2009). Discrimination of sleep-apnea-related decreases in the amplitude fluctuations of PPG signal in children by HRV analysis. IEEE Trans. Biomed. Eng 56 (4), 1005–1014. 10.1109/TBME.2008.2009340 19272873

[B28] Gold S. M. Zakowski S. G. Valdimarsdottir H. B. Bovbjerg D. H. (2004). Higher Beck depression scores predict delayed epinephrine recovery after acute psychological stress independent of baseline levels of stress and mood. Biol. Psychol 67, 261–273. 10.1016/j.biopsycho.2003.12.001 15294385

[B29] Greco A. Valenza G. Lanata A. Scilingo E. P. Citi L. (2016). cvxEDA: A convex optimization approach to electrodermal activity processing. IEEE Trans. Biomed. Eng 63 (4), 797–804. 10.1109/TBME.2015.2474131 26336110

[B30] Guillen-Riquelme A. Buela-Casal G. (2011). Psychometric revision and differential item functioning in the state trait anxiety inventory (STAI). Psicothema 23, 510–515. 21774907

[B31] Haufe S. Kim J. W. Kim I. H. Sonnleitner A. Schrauf M. Curio G. (2014). Electrophysiology-based detection of emergency braking intention in real-world driving. J. Neural Eng 11, 056011. 10.1088/1741-2560/11/5/056011 25111850

[B32] Heart rate variability (1996). Standards of measurement, physiological interpretation and clinical use. Task Force of the European society of cardiology and the north American society of pacing and electrophysiology. Circulation 93 (5), 1043–1065. PMID: 8598068. 8598068

[B33] Hellhammer D. H. Stone A. A. Hellhammer J. Broderick J. (2010). Measuring stress, in Encyclopedia of behavioral neuroscience. Editors Koob G. F. Le Moal M. Thompson R. (Oxford: Academic Press), 186–191.

[B34] Hellhammer D. H. Wüst S. Kudielka B. M. (2009). Salivary cortisol as a biomarker in stress research. Psychoneuroendocrinology 34, 163–171. 10.1016/j.psyneuen.2008.10.026 19095358

[B35] Hernando A. Lazaro J. Gil E. Arza Valdes A. Garzon-Rey J. Lopez-Anton R. (2016). Inclusion of respiratory frequency information in heart rate variability analysis for stress assessment. IEEE J. Biomed. Health Inf 20, 1016–1025. 10.1109/JBHI.2016.2553578 27093713

[B36] Het S. Rohleder N. Schoofs D. Kirschbaum C. Wolf O. T. (2009). Neuroendocrine and psychometric evaluation of a placebo version of the “trier social stress test”. Psychoneuroendocrinology 34, 1075–1086. 10.1016/j.psyneuen.2009.02.008 19307062

[B37] Holmes T. H. Rahe R. H. (1967). The social readjustment rating scale. J. Psychosom. Res 11, 213–218. 10.1016/0022-3999(67)90010-4 6059863

[B38] Jezova D. Makatsori A. Duncko R. Moncek F. Jakubek M. (2004). High trait anxiety in healthy subjects is associated with low neuroendocrine activity during psychosocial stress. Prog. Neuropsychopharmacol. Biol. Psychiatry 28, 1331–1336. 10.1016/j.pnpbp.2004.08.005 15588760

[B39] Kim H. G. Cheon E. J. Bai D. S. Lee Y. H. Koo B. H. (2018). Stress and heart rate variability: A meta-analysis and review of the literature. Psychiatry Investig 15 (3), 235–245. 10.30773/PI.2017.08.17 PMC5900369 29486547

[B40] Kudielka B. M. Hellhammer H. Kirschbaum C. Hellhammer D. H. Kirschbaum C. Harmon-Jones E. (2007). Ten years of research with the trier social stress test—Revisited, in Social neuroscience (New York, NY: Guilford Press), 512.

[B41] Lackner H. K. Papousek I. Batzel J. J. Roessler A. Scharfetter H. Hinghofer-Szalkay H. (2011). Phase synchronization of hemodynamic variables and respiration during mental challenge. Int. J. Psychophysiol 79, 401–409. 10.1016/j.ijpsycho.2011.01.001 21223982

[B42] Lázaro J. Gil E. Vergara J. M. Laguna P. (2014). Pulse rate variability analysis for discrimination of sleep-apnea-related decreases in the amplitude fluctuations of pulse photoplethysmographic signal in children. IEEE J. Biomed. Health Inf 18 (1), 240–246. 10.1109/JBHI.2013.2267096 24403422

[B43] Liu J. J. W. Ein N. Peck K. Huang V. Pruessner J. C. Vickers K. (2017). Sex differences in salivary cortisol reactivity to the trier social stress test (TSST): A meta-analysis. Psychoneuroendocrinology 82, 26–37. Epub 2017 Apr 24. PMID: 28486178. 10.1016/j.psyneuen.2017.04.007 28486178

[B44] Martínez J. P. Almeida R. Olmos S. Rocha A. P. Laguna P. (2004). A wavelet-based ECG delineator: Evaluation on standard databases. IEEE Trans. Biomed. Eng 51 (4), 570–581. 10.1109/TBME.2003.821031 15072211

[B45] Mateo J. Laguna P. (2003). Analysis of heart rate variability in the presence of ectopic beats using the heart timing signal. IEEE Trans. Biomed. Eng 50 (3), 334–343. 10.1109/TBME.2003.808831 12669990

[B46] Milagro J. Gil E. Garzón-Rey J. M. Aguiló J. Bailón R. (September 2017). Inspiration and expiration dynamics in acute emotional stress assessment, in Proceedings of the 2017 Computing in Cardiology (CinC). Rennes, France, IEEE, 10.22489/CinC.2017.261-411

[B47] Monk T. H. (1989). A Visual Analogue Scale technique to measure global vigor and affect. Psychiatry Res 27, 89–99. 10.1016/0165-1781(89)90013-9 2922449

[B48] Pisanski K. Kobylarek A. Jakubowska L. Nowak J. Walter A. Błaszczyński K. (2018). Multimodal stress detection: Testing for covariation in vocal, hormonal and physiological responses to Trier Social Stress Test. Horm. Behav 106, 52–61. Epub 2018 Sep 19. PMID: 30189213. 10.1016/j.yhbeh.2018.08.014 30189213

[B49] Posada-Quintero H. F. Florian J. P. Orjuela-Cañón A. D. Aljama-Corrales T. Charleston-Villalobos S. Chon K. H. (2016). Power spectral density analysis of electrodermal activity for sympathetic function assessment. Ann. Biomed. Eng 44 (10), 3124–3135. 10.1007/s10439-016-1606-6 27059225

[B50] Remor E. (2006). Psychometric properties of a European Spanish version of the perceived stress scale (PSS). Span. J. Psychol 9, 86–93. 10.1017/s1138741600006004 16673626

[B51] Rohleder N. Nater U. M. Wolf J. M. Ehlert U. Kirschbaum C. (2004). Psychosocial stress-induced activation of salivary alpha-amylase: An indicator of sympathetic activity? Ann. N. Y. Acad. Sci 1032, 258–263. 10.1196/annals.1314.033 15677423

[B52] Selye H. (1950). Allergy and the general adaptation syndrome. Int. Arch. Allergy Appl. Immunol 3, 267–278. 10.1159/000227975 13044322

[B53] Sequeira I. K. Longmire A. S. McKay N. J. (2021). Trier social stress test elevates blood pressure, heart rate, and anxiety, but a singing test or unsolvable anagrams only elevates heart rate, among healthy young adults. Psych 3 (2), 171–183. 10.3390/psych3020015

[B54] Spielberger C. D. (2010). State-trait anxiety inventory, in The corsini encyclopedia of psychology. Editor Stricke G. (Hoboken, NJ, USA: John Wiley & Sons), 4–6. 10.1002/9780470479216.corpsy0943

[B55] Stetter F. Kupper S. (2002). Autogenic training: A meta-analysis of clinical outcome studies. Appl. Psychophysiol. Biofeedback 27 (1), 45–98. 10.1023/A:1014576505223 12001885

[B56] Takai N. Yamaguchi M. Aragaki T. Eto K. Uchihashi K. Nishikawa Y. (2004). Effect of psychological stress on the salivary cortisol and amylase levels in healthy young adults. Arch. Oral Biol 49, 963–968. 10.1016/j.archoralbio.2004.06.007 15485637

[B57] Urwyler S. A. Schuetz P. Sailer C. Christ-Crain M. (2015). Copeptin as a stress marker prior and after a written examination – The CoEXAM study. Stress 18, 134–137. 10.3109/10253890.2014.993966 25472823

[B58] Varon C. Lazaro J. Bolea J. Hernando A. Aguilo J. Gil E. (2019). Unconstrained estimation of HRV indices after removing respiratory influences from heart rate. IEEE J. Biomed. Health Inf 23, 2386–2397. 10.1109/JBHI.2018.2884644 30507541

[B59] Vinkers C. H. Penning R. Hellhammer J. Verster J. C. Klaessens J. H. Olivier B. (2013). The effect of stress on core and peripheral body temperature in humans. Stress 16, 520–530. 10.3109/10253890.2013.807243 23790072

[B60] Vlemincx E. Taelman J. De Peuter S. Van Diest I. Van den Bergh O. (2011). Sigh rate and respiratory variability during mental load and sustained attention. Psychophysiology 48, 117–120. 10.1111/j.1469-8986.2010.01043.x 20536901

[B61] Wong M. Y. Pickwell-MacPherson E. Zhang Y. T. Cheng J. C. (2011). The effects of pre-ejection period on post-exercise systolic blood pressure estimation using the pulse arrival time technique. Eur. J. Appl. Physiol 111 (1), 135–144. 10.1007/s00421-010-1626-0 20824282

[B62] Yamanaka Y. Motoshima H. Uchida K. (2019). Hypothalamic-pituitary-adrenal axis differentially responses to morning and evening psychological stress in healthy subjects. Neuropsychopharmacol. Rep 39, 41–47. 10.1002/npr2.12042 30480877 PMC7292277

